# Cardiovascular autonomic neuropathy and the risk of diabetic kidney disease

**DOI:** 10.3389/fendo.2024.1462610

**Published:** 2024-09-12

**Authors:** Injeong Cho, Seohyun Lim, Minjae Kwon, Seung Min Chung, Jun Sung Moon, Ji Sung Yoon, Kyu Chang Won

**Affiliations:** ^1^ College of Medicine, Yeungnam University, Daegu, Republic of Korea; ^2^ Division of Endocrinology and Metabolism, Department of Internal Medicine, Yeungnam University College of Medicine, Daegu, Republic of Korea

**Keywords:** diabetic nephropathies, autonomic nervous system diseases, diabetic neuropathies, diabetes complications, diabetes mellitus

## Abstract

**Background:**

Cardiovascular autonomic neuropathy (CAN) is known to affect patients with diabetes mellitus (DM) and cause adverse renal outcomes. We aimed to analyze the association between CAN and diabetic kidney disease (DKD).

**Method:**

We enrolled 254 DM patients (mean age, 56.7 ± 15.2 years; male: female ratio, 1.17:1) with 19 (7.5%) type 1 DM patients and 235 (92.5%) type 2 DM patients. All patients had undergone cardiovascular autonomic function tests between January 2019 and December 2021 in a tertiary hospital in Korea. Cardiovascular autonomic neuropathy was categorized as normal, early, or definite after measuring three heart rate variability parameters. Diabetic kidney disease refers to a persistently elevated urinary albumin-creatinine ratio (uACR ≥30 mg/g) or reduced estimated glomerular filtration rate (eGFR <60 mL/min/1.73 m^2^). Logistic and Cox regression analyses were performed.

**Results:**

Patients with elevated uACR (n=107) and reduced eGFR (n=32) had a higher rate of definite CAN. After adjusting for covariates, definite CAN was associated with elevated uACR (OR=2.4, 95% CI 1.07-5.36) but not with reduced eGFR (OR=3.43, 95% CI 0.62-18.90). A total of 94 patients repeated uACR measurements within 2 years (mean follow-up, 586.3 ± 116.8 days). Both definite and early CAN were independent risk factors for elevated uACR (HR=8.61 and 8.35, respectively; both *p*<0.05). In addition, high-density lipoprotein cholesterol, ACE inhibitors/angiotensin receptor blockers and glucagon-like peptide-1 receptor agonists were independent protective factors for elevated uACR (HR=0.96, 0.25, and 0.07, respectively; all *p*<0.05).

**Conclusion:**

Cardiovascular autonomic neuropathy is a potential indicator of DKD. Comprehensive management of DKD in the early stages of CAN may prevent microalbuminuria.

## 1 Introduction

The prevalence of diabetes mellitus (DM) has been increasing in the past 4 years and is estimated to be 16.7% in 2020 among South Korean adults over 30 years of age (1). Glucose control aims to prevent microvascular and macrovascular complications (2). The risks of end-stage renal disease, myocardial infarction, and heart failure in patients with DM are 4.95, 1.59, and 1.51 times higher than those in patients with normoglycemia, respectively (1). Diabetic kidney disease (DKD) is the main cause of end-stage renal disease and is associated with increased cardiovascular disease and mortality (3). To reduce the risk of DKD progression and cardiovascular disease, comprehensive management with evidence-based medication is recommended (2, 4, 5).

Persistent hyperglycemia also damages autonomic nerve fibers and induces abnormal heart rate and blood pressure responses, known as cardiovascular autonomic neuropathy (CAN) (6). Cardiovascular autonomic neuropathy affects one-fourth of type 1 DM and one-third of type 2 DM cases and increases the risk of silent myocardial ischemia and sudden cardiac death (6). Interestingly, heart rate variability was reported to be related to the progression of end-stage renal disease (7–9), which may result from alterations in renal hemodynamics (10). However, whether the presence of CAN can predict the early stages of DKD has not been well explored.

Therefore, we aimed to analyze the prevalence of normal, early, and definite CAN among patients with DM, as well as to explore the cross-sectional and longitudinal association between CAN and DKD. In addition, we explored the benefits of comprehensive DM management in reducing DKD, regardless of CAN.

## 2 Methods

### 2.1 Study population

**Figure 1:**
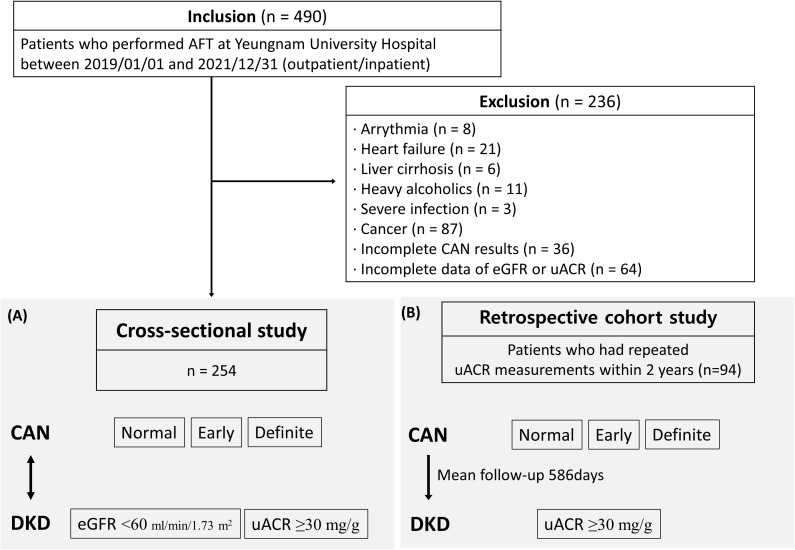
Schematic of the study population with **(A)** cross-sectional analysis for 254 patients and **(B)** retrospective cohort study for 94 patients who underwent repeated uACR measurements within 2 years.

This study screened 490 patients with DM who underwent cardiovascular autonomic function tests (AFT) between January 1, 2019, and December 31, 2021, at Yeungnam University Hospital. Of these patients, eight patients with arrhythmia, 21 with heart failure, 6 with liver cirrhosis, 11 suffering from severe alcoholism (alcohol intake over 20 g/day in females and 30 g/day in males, or diagnosed with alcohol-induced liver disease) (11), 3 with severe infection, 87 with cancer, 36 with incomplete AFT results, and 64 with incomplete data of estimated glomerular filtration rate (eGFR) or urinary albumin-creatinine ratio (uACR) were excluded. Finally, 254 patients were included in the cross-sectional analysis (**Figure 1A**). There were 19 (7.5%) patients with type 1 DM and 235 (92.5%) with type 2 DM. In addition, a retrospective cohort study was designed for 94 patients who underwent repeated uACR measurements within 2 years (**Figure 1B**). The effect of normal, early, and definite CAN for DKD was assessed in each study design. This study was approved by the Institutional Review Board of Yeungnam University Hospital (IRB No. 2023-10-007) and exempted from informed consent. All methods were performed in accordance with the relevant guidelines and regulations.

### 2.2 Data collection

The demographic and clinical data of the patients included age, sex, type and duration of DM, systolic and diastolic blood pressure (BP), body mass index (BMI), smoking status, alcohol consumption, history of hypertension, and cardiovascular disease. The duration of DM was calculated as the period from the initial DM diagnosis to the date of AFT. Previous and current smokers were categorized as smokers. Hypertension was defined as systolic blood pressure ≥ 140 mmHg or diastolic blood pressure ≥ 90 mmHg or taking antihypertensive medication. Cardiovascular disease was defined as a history of myocardial infarction, peripheral arterial occlusive disease, or cerebrovascular disease.

Laboratory data included glycated hemoglobin (HbA1c), fasting glucose, fasting insulin, aspartate aminotransferase (AST), alanine aminotransferase (ALT), eGFR, uACR, total cholesterol, triglycerides, high-density lipoprotein (HDL) cholesterol, and low-density lipoprotein (LDL) cholesterol. The homeostasis model assessment of insulin resistance (HOMA-IR) and β-cell function (HOMA-β) were calculated as follows: (glucose [mg/dL] × insulin [μU/mL]/405) and (360 × insulin [μIU/mL])/(glucose [mg/dL] – 63), respectively (12).

Medications recommended for the comprehensive management of DKD were collected (5): insulin, angiotensin-converting enzyme inhibitors (ACEi), angiotensin II receptor blockers (ARB), sodium–glucose cotransporter-2 inhibitors (SGLT2i), glucagon-like peptide-1 receptor agonists (GLP-1RA), and statins. Data were collected based on the prescription history 3 months before and after the date of AFT assessment.

### 2.3 Assessment of CAN

Cardiovascular AFT was performed using AFT-800 (MEDICORE, Korea). Caffeine intake, smoking, alcohol consumption, and antihypertensive medication use were prohibited for 12 h before the test since caffeine, nicotine, and antihypertensive drugs can influence autonomic function (e.g., blood pressure) (13). Vigorous exercise, which can result in variations in heart rate, was restricted to 24 h before the test (14, 15).

We adopted the Ewing method and performed three standardized tests to assess heart rate variability: the Valsalva maneuver, heart rate response from lying to standing positions and to deep breathing (16). These tests reflect parasympathetic and cardiovagal functions with high sensitivity, specificity, and reproducibility (17). Heart-rate variability assessed by the three tests has a sensitivity, specificity, positive predictive value, and negative predictive value of more than 90%, and has been proven to be a simple method to predict cardiovascular complications and mortality (18). The detailed inspection method has been published elsewhere (16).

The heart rate response to the Valsalva maneuver was calculated using the Valsalva ratio (longest R-R interval after the Valsalva maneuver/shortest R-R interval during the Valsalva maneuver). The Valsalva ratio of ≥1.21, 1.11–1.20, and ≤1.10 was categorized as normal, borderline, and abnormal, respectively (16). The heart rate response from lying to standing was calculated using a 30:15 ratio (longest R-R interval around 30^th^ beat/the shortest R-R interval around the 15^th^ beat on the electrocardiogram). The 30:15 ratio of ≥1.04, 1.01–1.03, and ≤1.00 was categorized as normal, borderline, and abnormal, respectively (16). The heart rate response to deep breathing was calculated as the median value of the difference between the maximum and minimum heart rates after six cycles of even breathing for 1 min. The value of ≥15, 11-14, and ≤10 was categorized into normal, borderline, and abnormal, respectively (16).

The results of the three tests were converted to 0, 0.5, and 1 point for normal, borderline, and abnormal conditions, respectively. The total points were calculated and classified into normal CAN (0–0.5 point), early CAN (1 point), and definite CAN (≥1.5 point) (19).

### 2.4 Assessment of DKD

The renal function was assessed using eGFR and uACR. The eGFR was calculated using Modification of Diet in Renal Disease formula: = 175 × (SCr)^-1.154^ x (age)^-0.203^ × 0.742 [if female]. DKD was defined as persistently elevated uACR (≥30 mg/g), persistently reduced eGFR (<60 mL/min/1.73 m^2^), or both, for longer than 3 months in the presence of longstanding DM in accordance with the current KDIGO guidelines (5). Both reduced eGFR and elevated uACR were designated as outcomes of this study.

### 2.5 Sample size calculation

The sample size estimation for DKD was performed using previously published data. A previous prospective study demonstrated that among 204 patients with type 2 DM, the prevalence of elevated uACR in patients with CAN was 34% (8). We projected a sample size of 165 to obtain a power of 0.80 with an alpha of 0.05 (20). Therefore, our study had adequate power to evaluate DKD according to CAN. The equation for sample size calculations is presented in **Supplementary Data**.

### 2.6 Statistical analysis

All statistical analyses were performed using the SPSS Statistics version 27(IBM Corp. Armonk, NY, USA). We used one-way ANOVA or independent sample t-test to compare continuous variables and the chi-square test to compare categorical variables. Logistic regression analysis was performed to investigate the risk factors in patients for reduced eGFR and elevated uACR. Cox regression and Kaplan–Meier curve analyses were performed to investigate the longitudinal impact of definite CAN on elevated uACR. Follow-up duration was designated as interval between uACR measurements. Statistical significance was set at p<0.05.

## 3 Results

### 3.1 Baseline characteristics

A total of 254 participants were included in the analysis. The average age of the subjects was 56.7 ± 15.2 years, and the M:F ratio was 1.17:1. There were 19 (7.5%) patients with type 1 DM and 235 (92.5%) with type 2 DM, and the baseline characteristics of these two groups are summarized in **Supplementary Table 1**. According to AFT results, patients with normal, early, and definite CAN accounted for 21.3% (n=54), 35.8% (n=91), and 42.9% (n=109), respectively. The baseline characteristics of patients with normal, early, and definite CAN are summarized in **Table 1**. In the sequential order of normal, early, and definite CAN, patients were older, had a longer duration of diabetes, had lower eGFR, and had a higher proportion taking ACEi/ARB (all p<0.05). Alcohol consumption was the highest among patients with early CAN (p<0.05). The uACR was the highest in definite CAN; however, this difference was not significant (p=0.207). The use of insulin, SGLT2i, GLP-1RA, and statins was not significantly different among the groups (all p>0.05).

**Table 1:** Baseline characteristics according to CAN status.

	Total	CAN			*p*-value
		Normal	Early	Definite	
**N (%)**	254	54 (21.3)	91 (35.8)	109 (42.9)	
**Age, years**	56.7 ± 15.2	45.3 ± 15.7	57.4 ± 13.4	61.8 ± 13.3	<0.001
Sex, n(%)					
**Men**	137 (53.9)	35 (64.8)	49 (53.8)	53 (48.6)	0.149
**Women**	117 (46.1)	19 (35.2)	42 (46.2)	56 (51.4)	
Type of DM, n(%)					
**Type 1 DM**	19 (7.5)	5 (9.3)	8 (8.8)	6 (5.5)	0.578
**Type 2 DM**	235 (92.5)	49 (90.7)	83 (91.2)	103 (94.5)	
**Duration of DM, years**	7.3 ± 8.3	4.1 ± 5.7	6.4 ± 7.4	9.6 ± 9.4	<0.001
**Systolic BP, mmHg**	128 ± 19.7	128.5 ± 19.2	124.8 ± 19.7	130.6 ± 19.7	0.124
**Diastolic BP, mmHg**	78.9 ± 12.1	82.5 ± 12.4	78.3 ± 11.4	77.6 ± 12.3	0.051
**BMI, kg/m^2^**	24.7 ± 4.6	25.6 ± 5.1	24.2 ± 4.1	24.7 ± 4.6	0.219
**Smoking, n (%)**	101 (41.1)	25 (47.1)	36 (41.3)	40 (37.7)	0.411
**Alcohol, n (%)**	104 (42.3)	20 (37.8)	40 (45.9)	44 (41.5)	0.041
**Hypertension, n (%)**	139 (54.7)	26 (48.1)	46 (50.5)	67 (61.5)	0.167
**Cardiovascular disease, n (%)**	48 (19.0)	8 (14.8)	13 (14.3)	27 (25.0)	0.108
**HbA1c, %**	10.2 ± 2.7	10.9 ± 2.5	10.1 ± 3.0	10.0 ± 2.6	0.109
**Fasting glucose, mg/dL**	187.2 ± 71.3	192.5 ± 59.2	179.3 ± 65.7	191.1 ± 81.7	0.478
**Fasting Insulin, uIU/mL**	11.4 ± 10.5	11.9 ± 11.1	10.7 ± 8.2	11.8 ± 11.8	0.753
**HOMA IR**	7.2 ± 29.3	14.1 ± 59.9	5.0 ± 3.7	5.1 ± 4.4	0.161
**HOMA B**	58.9 ± 173.2	43.4 ± 47.5	47.3 ± 50.4	77.8 ± 263.7	0.413
**AST, IU/L**	27.8 ± 20.0	33.3 ± 32.3	25.6 ± 13.1	26.9 ± 16.2	0.068
**ALT, IU/L**	31.7 ± 22.0	34.2 ± 26.3	29.7 ± 18.5	32.2 ± 22.5	0.469
**Total cholesterol, mg/dL**	179.8 ± 84.5	204.0 ± 147.5	173.8 ± 54.2	172.2 ± 53.5	0.56
**Triglyceride, mg/dL**	188.8 ± 184.6	221.7 ± 218.1	188.7 ± 204.9	171.4 ± 141.5	0.27
**HDL cholesterol, mg/dL**	47.4 ± 14.4	44.4 ± 12.7	48.3 ± 14.4	48.2 ± 15.1	0.228
**LDL cholesterol, mg/dL**	92.0 ± 44.3	97.6 ± 42.6	90.7 ± 46.1	90.1 ± 43.8	0.572
**eGFR, mL/min/1.73m^2^**	103.6 ± 38.5	121.8 ± 36.6	102.0 ± 31.7	95.9 ± 41.9	<0.001
**uACR, mg/g**	122.6 ± 349.7	90.8 ± 222.9	82.9 ± 234.0	171.5 ± 460.7	0.207
**Insulin, n (%)**	204 (80.6)	42 (79.2)	74 (81.3)	88 (80.7)	0.954
**ACEi/ARB, n (%)**	108 (42.7)	18 (34.0)	33 (36.3)	57 (52.3)	0.026
**SGLT2i, n (%)**	63 (24.9)	17 (32.1)	19 (20.9)	27 (24.8)	0.325
**GLP-1RA, n (%)**	10 (4.0)	1 (1.9)	2 (2.2)	7 (6.4)	0.214
**Statin, n (%)**	179 (70.8)	35 (66.0)	60 (65.9)	84 (77.1)	0.158

### 3.2 Association between clinical parameters and DKD

**Figure 2:**
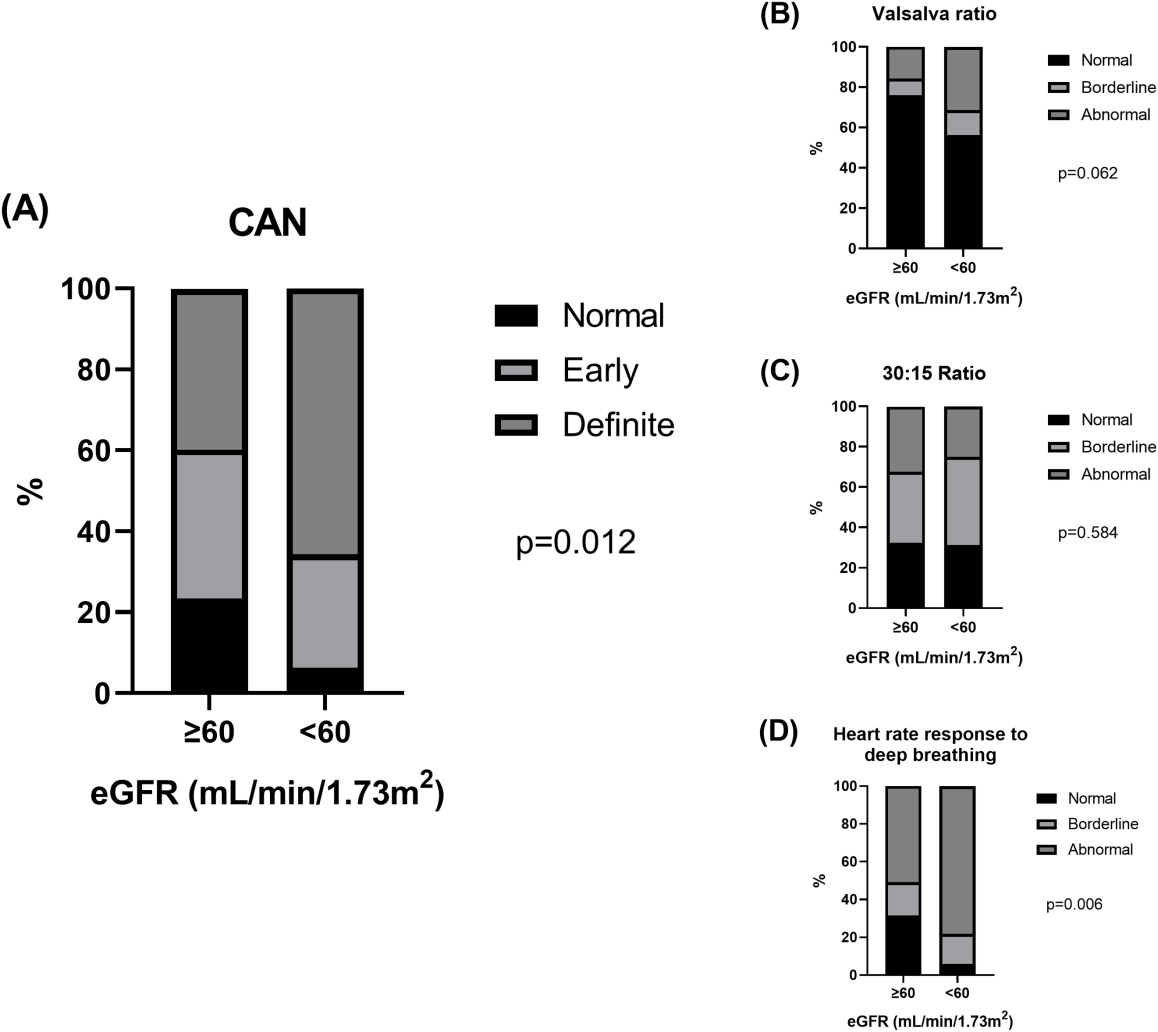
The proportion of **(A)** normal, early, and definite cardiovascular autonomic neuropathy (CAN) and **(B-D)** normal, borderline, and abnormal heart rate responses to Valsalva maneuver (Valsalva ratio), lying to standing (30:15 ratio), and deep breathing according to eGFR levels.

Patients’ characteristics between normal (≥60 mL/min per 1.73 m^2^; n=222 [87.4%]) and reduced eGFR (<60 mL/min per 1.73 m^2^; n=32 [12.6%]) are summarized in **Supplementary Table 2**. Compared with those with normal eGFR, patients with reduced eGFR were older, had a longer duration of diabetes, and had a higher proportion of comorbid hypertension and use of ACEi/ARB and statins (all p<0.05). The level of HDL cholesterol level was lower and that of uACR was higher in patients with reduced eGFR than in those with normal eGFR (both p<0.05). The proportion of normal, early, and definite CAN were significantly different between normal eGFR (23.4%, 36.9%, and 39.6%, respectively; p=0.012; **Figure 2A**) and reduced eGFR (6.3%, 28.1%, and 65.6%, respectively; p=0.012; **Figure 2A**). Compared with patients with normal eGFR, patients with reduced eGFR had a significantly higher proportion of abnormal heart rate responses to deep breathing (p=0.006; **Figure 2D**), whereas no difference was observed in the Valsalva (p=0.062; **Figure 2B**) and 30:15 ratios (p=0.584; **Figure 2C**).

**Figure 3:**
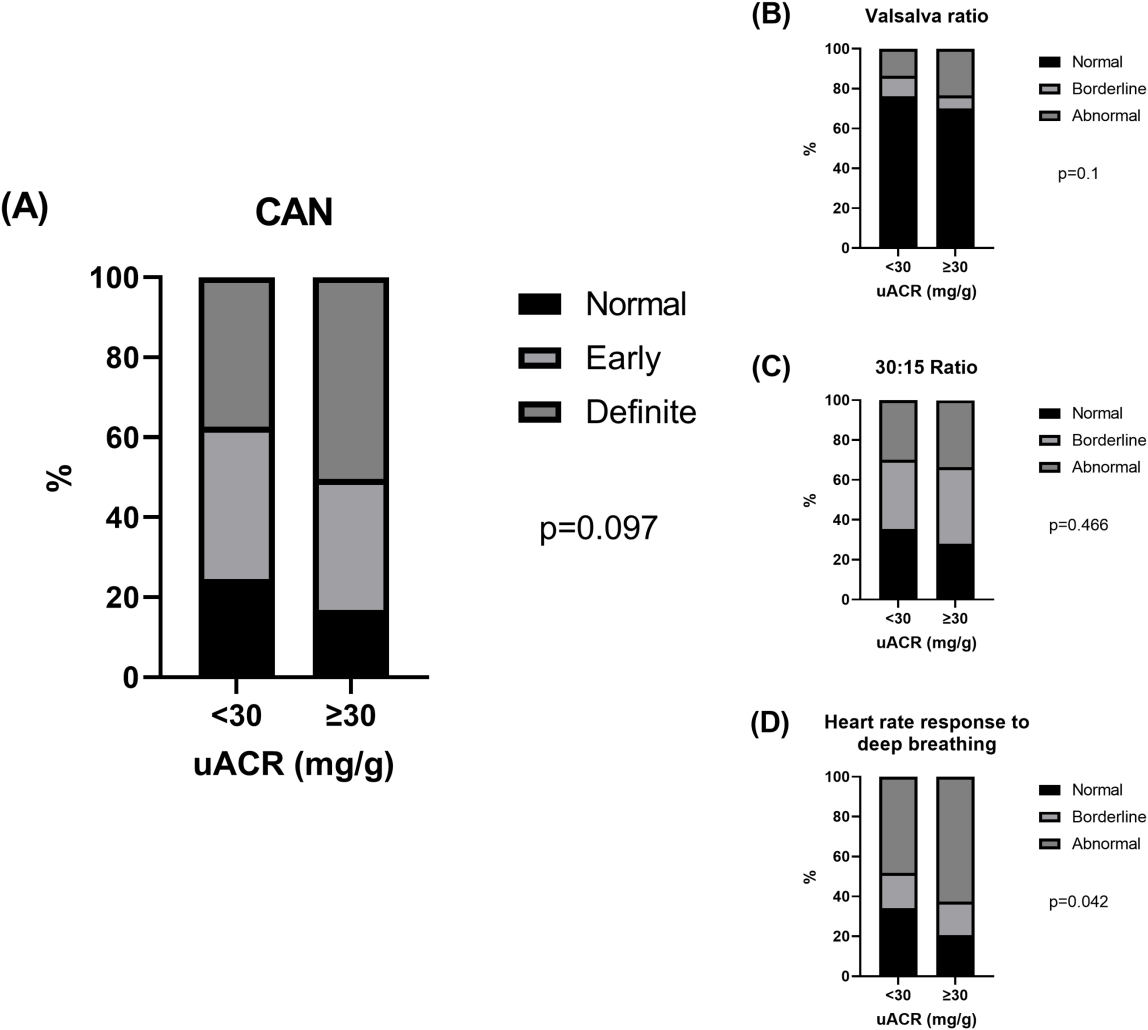
The proportion of **(A)** normal, early, and definite CAN and **(B-D)** normal, borderline, and abnormal heart rate responses to Valsalva maneuver (Valsalva ratio), lying to standing (30:15 ratio), and deep breathing according to uACR levels.

Patients’ characteristics between normoalbuminuric (uACR <30 mg/g; n=147 [57.9%]) and elevated uACR (≥30 mg/g; n=107[42.1%]) are summarized in **Supplementary Table 3**. Compared with those with normoalbuminuria, patients with elevated uACR had higher levels (or proportion) of systolic BP, diastolic BP, triglycerides, and comorbid hypertension (all p<0.05). In contrast, HDL cholesterol levels were lower in patients with elevated uACR than in those with normoalbuminuria (p<0.05). The normal, early, and definite CAN accounted for 24.5%, 38.1%, and 37.4% of patients with normoalbuminuria and 16.8%, 32.7%, and 50.5% of those with elevated uACR, respectively (p=0.097; **Figure 3A**). The Valsalva (p=0.100; **Figure 3B**) and 30:15 ratios (p=0.466; **Figure 3C**) did not differ between normal and elevated uACR, whereas the abnormal heart rate response to deep breathing was significantly higher in patients with elevated uACR than in those with normoalbuminuria (p=0.042; **Figure 3D**).

### 3.3 CAN and risk of DKD

Logistic regression analysis was performed to explore the risk factors for reduced eGFR (<60 mL/min/1.73 m2) and elevated uACR (≥30 mg/g). Prior to the analysis, a correlation test was conducted (**Supplementary Table 4**). In the univariate analysis (**Supplementary Table 5**), older age, longer duration of DM, presence of hypertension, high triglyceride levels, low HDL cholesterol levels, and use of ACEi/ARBs or statins were significant risk factors for reduced eGFR or elevated uACR (all p<0.05). In addition, definite CAN was a significant risk factor for reduced eGFR (OR=6.2, 95% CI 1.4–27.53, p=0.016) and a possible risk factor for elevated uACR with marginal significance (OR=1.96, 95% CI 1.0–3.87, p=0.051). Multivariate analysis was performed after adjusting for covariates which was significant risk factors in the univariate analysis (**Table 2**): model 1 was adjusted for age and sex; model 2 was adjusted for model 1 + duration of DM, BMI, and hypertension; model 3 was adjusted for model 2 + HbA1c, triglyceride, and HDL cholesterol; and model 4 was adjusted for model 3 + ACEi/ARB and statin. After adjustments, definite CAN had no significant effect on reduced eGFR (OR=3.43, 95% CI 0.62–18.90, p=0.157), whereas it was a significant risk factor for elevated uACR (OR=2.4, 95% Cl 1.07–5.36, p=0.034).

**Table 2:** The cross-sectional impact of CAN on diabetic kidney disease.

OR (95% CI, *p-*value)				
eGFR <60 mL/min/1.73 m^2^				
	model 1	model 2	model 3	model 4
**Normal CAN**	1 (ref)	1 (ref)	1 (ref)	1 (ref)
**Early CAN**	1.71 (0.34-8.62, 0.513)	2.15 (0.41-11.43, 9.368)	2.75 (0.49-15.41, 0.250)	2.68 (0.45-15.84, 0.276)
**Definite CAN**	3.12 (0.66-14.78, 0.151)	3.11 (0.63-15.47, 0.166)	4.08 (0.77-21.67, 0.099)	3.43 (0.62-18.90, 0.157)
uACR ≥30 mg/g				
	model 1	model 2	model 3	model 4
**Normal CAN**	1 (ref)	1 (ref)	1 (ref)	1 (ref)
**Early CAN**	1.27 (0.60-2.68, 0.527)	1.4 (0.65-3.02, 0.385)	1.69 (0.76-3.74, 0.200)	1.68 (0.75-3.76, 0.204)
**Definite CAN**	2.04 (0.96-4.31, 0.064)	2.02 (0.94-4.36, 0.072)	2.31 (1.04-5.12, 0.040)	2.4 (1.07-5.36, 0.033)

Model 1: adjusted for sex and age.

Model 2: adjusted for model 1 + duration of DM, BMI, hypertension.

Model 3: adjusted for model 2 + HbA1c, triglyceride, HDL cholesterol.

Model 4: adjusted for model 3 + ACEi/ARB, Statin.

A total of 94 patients who had repeated uACR measurements within 2 years were followed up to explore whether definite CAN longitudinally increased the risk of elevated uACR (**Table 3**). The patients were followed up for 586.3 ± 116.8 days. The risk of normal, early, and definite CAN on elevated uACR was visualized using a Kaplan–Meier curve. Early and definite CAN were associated with an earlier elevation of uACR compared with normal CAN (log-rank p=0.051; **Supplementary Figure 1**). Cox regression analysis was performed, and the results are presented in **Table 3**. In addition to the covariates considered in the logistic regression analysis, uACR levels at baseline and use of SGLT2i and GLP1-RA, evidence-based medications for renal protection, were adjusted. After adjustments, BMI (HR = 1.18, 95% CI 1.02–1.36, p=0.025), early CAN (HR=8.35, 95% CI 1.46–47.88, p=0.017), and definite CAN (HR=8.61, 95% CI 1.53–48.29, p=0.014) were independent risk factors for elevated uACR. In contrast, HDL cholesterol (HR=0.96, 95% CI 0.91–1.00, p=0.044), ACEi/ARB (HR=0.25, 95% CI 0.08–0.84), and GLP-1RA (HR=0.07, 95% CI 0.01–0.7, p=0.023) were independent protective factors for elevated uACR.

**Table 3:** The longitudinal impact of CAN and variables on elevated uACR.

		adjusted HR (95%CI)	adjusted *p*-value
**Age**		1 (0.96,1.05)	0.892
**Men vs. Women**		2.85 (0.99,8.26)	0.053
**Duration of DM**		1.03 (0.95,1.1)	0.509
**BMI**		1.18 (1.02,1.36)	0.025
**Hypertension**		1.62 (0.66,3.96)	0.292
**HbA1c**		0.91 (0.7,1.2)	0.522
**Triglyceride**		1 (1,1)	0.598
**HDL cholesterol**		0.96 (0.91,1)	0.044
**uACR**		1 (1,1)	0.785
**ACEi/ARB**		0.25 (0.08,0.84)	0.025
**Statin**		1.2 (0.36,3.95)	0.769
**SGLT2i**		0.94 (0.33,2.66)	0.9
**GLP-1RA**		0.07 (0.01,0.7)	0.023
**CAN**	normal		
	early	8.35 (1.46,47.88)	0.017
	definite	8.61 (1.53,48.29)	0.014

## 4 Discussion

The primary aim of this study was to determine whether CAN is associated with DKD. This study demonstrated that CAN was an independent risk factor for DKD, particularly elevated uACR (≥30 mg/g). Cross-sectionally, definite CAN increased the risk of elevated uACR by 2.4-fold, and longitudinally, early, and definite CAN increased the risk of elevated uACR by 8.3- and 8.6-fold, respectively. In addition to early and definite CAN, BMI was an independent risk factor, whereas high HDL levels, ACEi/ARBs, and GLP-1RA were independent protective factors for elevated uACR. CAN is a clinically important form of diabetic neuropathy that can be easily assessed by evaluating heart rate variability after deep breathing, the Valsalva maneuver, and postural changes (17). CAN has a significant impact on the occurrence of cardiovascular diseases, such as heart failure, fatal arrhythmia, and sudden cardiac death, in patients with diabetes (21, 22). This is because the sympathetic and parasympathetic nerves in the autonomic nervous system control the cardiac output, myocardial contraction, and contraction/relaxation of the vessels (23). The prevalence of CAN increases with age and duration of diabetes and is high in individuals with obesity, smoking addiction, and poor glycemic control (23, 24). Cumulative data suggest that CAN precede insulin resistance and inflammation cascade of DM (23, 25) and may present in the early stages of DKD (26). Therefore, heart rate variability can be used as a predictor of DKD.

The earliest manifestations of diabetic CAN are parasympathetic denervation and a compensatory increment in cardiac sympathetic tone (21). CAN-related sympathetic activation alters glomerular hemodynamics and circadian rhythms of blood pressure, inducing albuminuria and DKD progression (25). In this study, patients with decreased eGFR and elevated uACR had a higher rate of abnormal heart rate responses to deep breathing. Another study reported markedly reduced 24-h vagal activity in patients with elevated uACR (27). Recently, parasympathetic innervation of the renal artery and pelvis, for which no significant anatomical evidence previously exists, has been found in mice (28). These results suggest that CAN-related parasympathetic alterations may also contribute to DKD; however, further studies are warranted.

Few studies have examined the association between CAN and DKD. Heart rate variability predicts a decrease in eGFR among patients with type 1 DM after 10–11 years (29) and among those with type 2 DM after 2.5 and 10 years (8, 9). Moreover, heart rate variability is a significant predictor of cardiovascular disease in patients with DKD (30). Low heart rate variability is associated with hospitalization due to end-stage renal disease and chronic kidney disease (7). It is also strongly associated with early progressive renal decline in type 1 DM, measured by early GFR loss and advanced CKD (31). Another study reported that small pupil size at baseline, but not heart rate variability, was associated with microalbuminuria at 12 years of follow-up (32). Overall, the findings of the present study align with those reported in previous studies that have shown a close association between heart rate variability and either DM or adverse renal outcomes.

Notably, our study showed that heart rate variability has a significant relationship with an increase in uACR, which precedes eGFR decline (33), both cross-sectionally and longitudinally at 2 years of follow-up. In addition to definite CAN being a significant risk factor for elevated uACR, elevated uACR can be prospectively predicted from an early CAN stage. Furthermore, we confirmed that management of BMI and HDL cholesterol and treatment with ACEi/ARB and GLP-1RA can prevent microalbuminuria. It might be beneficial to undergo AFT in patients with DM and promptly start comprehensive management of DKD if early or definite CAN is present.

This study has certain limitations. First, it was a single center retrospective study conducted among people from the same ethnic group. Second, the cross-sectional analysis of the relationship between CAN and DKD makes it difficult to ascertain causality. For instance, the usage of ACEi/ARB and statin seemed to be a risk factor for reduced eGFR, however, it should be interpreted that patients with reduced eGFR were already taking these medications. Third, there were many losses during follow-up for cohort study and the follow-up period was relatively short (<2 years). Finally, due to the limited sample size of the type 1 DM cohort, the study was unable to establish whether CAN is a distinct predictor of diabetic nephropathy in type 1 DM compared to type 2 DM patients.

However, this study has strength that it revealed a causal relationship between CAN and DKD using longitudinal analysis after adjusting for a wide range of potential confounders. In addition, we confirmed the benefits of treatment with ACEi/ARB and GLP-1RA, along with weight reduction and elevation of HDL cholesterol levels, to prevent microalbuminuria regardless of CAN.

In conclusion, CAN is associated with DKD in patients with DM and predicts elevated uACR independent of possible confounders. CAN can be used as a potential risk factor to identify patients at increased risk of DKD, and early comprehensive management of DKD is necessary in the early stages of CAN.

## Data Availability

The raw data supporting the conclusions of this article will be made available by the authors, without undue reservation.
