# Supplementary material for: Cardiovascular autonomic neuropathy and the risk of diabetic kidney disease
Source: Front Endocrinol (Lausanne). 2024 Sep 12;15:1462610. doi: 10.3389/fendo.2024.1462610 (PMC11424464; doi:10.3389/fendo.2024.1462610)
Supplement: Supplementary file 1 [file DataSheet1.pdf]

Supplementary data. The equation for sample size calculation

$$N = \frac{p_0 q_0 \left\{ z_{1-\alpha/2} + z_{1-\beta} \sqrt{p_1 q_1 / p_0 q_0} \right\}^2}{(p_1 - p_0)^2}$$

q_0 = 1-p_0

q_1= 1-p_1

p_0 = proportion (incidence) of population

p_1 = proportion (incidence) of study group

N = sample size for study group

α = probability of type I error (usually 0.05)

β = probability of type II error (usually 0.2)

z = critical Z value for a given α or β

Supplementary Fig. 1. Kaplan–Meier curve for 2-year risk of CAN on elevated uACR

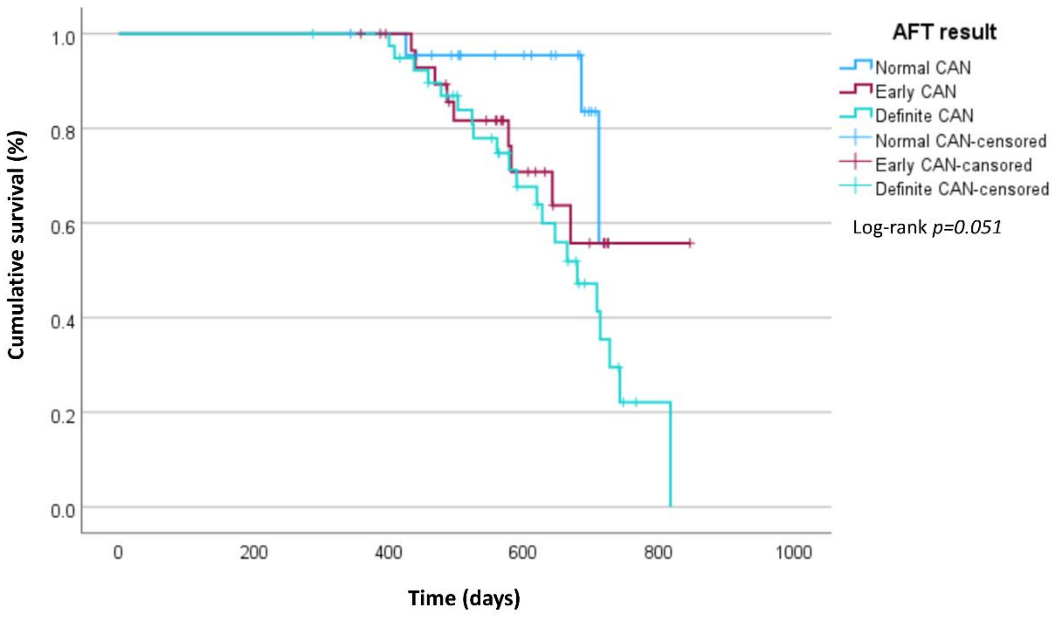

Supplementary Table 1. Baseline characteristics according to DM type

	Total	Type 1 DM	Type 2 DM	*p*-value
**n (%)**	254	19 (7.5)	235 (92.5)	
**Age, years**	56.7 ± 15.2	40.8 ± 16.1	58.0 ± 14.4	<0.001
**Sex, n(%)**				0.042
**Men**	137 (53.9)	6 (31.6)	131 (55.7)	
**Women**	117 (46.1)	13 (68.4)	104 (44.3)	
**Duration of DM, years**	7.3 ± 8.3	6.0 ± 7.1	7.4 ± 8.4	0.454
**Systolic BP, mmHg**	128 ± 19.7	127.1 ± 20.2	128.1 ± 19.7	0.829
**Diastolic BP, mmHg**	78.9 ± 12.1	79.6 ± 10.8	78.9 ± 12.2	0.788
**BMI, kg/m^2**	24.7 ± 4.6	21.9 ± 4.7	24.9 ± 4.5	0.005
**Smoking, n (%)**	101 (41.1)	5 (27.8)	106 (42.1)	0.458
**Alcohol, n (%)**	104 (42.3)	3 (16.7)	101 (44.3)	0.071
**Hypertension, n (%)**	139 (54.7)	4 (21.1)	135 (57.4)	0.002
**Cardiovascular disease, n (%)**	48 (19.0)	0 (0)	48 (20.5)	0.028
**Baseline HbA1c, %**	10.2 ± 2.7	11.0 ± 2.6	10.1 ± 2.7	0.175
**Fasting glucose, mg/dL**	187.2 ± 71.3	211.2 ± 74.0	185.4 ± 71.0	0.175
**Fasting Insulin, uIU/mL**	11.4 ± 10.5	5.1 ± 3.7	11.9 ± 10.6	<0.001
**HOMA IR**	7.2 ± 29.3	2.8 ± 2.4	7.5 ± 30.3	0.565
**HOMA B**	58.9 ± 173.2	15.9 ± 16.6	61.9 ± 178.8	0.337
**AST, IU/L**	27.8 ± 20.0	16.7 ± 6.6	28.7 ± 20.5	<0.001
**ALT, IU/L**	31.7 ± 22.0	17.8 ± 6.5	32.9 ± 22.5	<0.001
**Total cholesterol, mg/dL**	179.8 ± 84.5	193.6 ± 46.8	178.7 ± 86.7	0.473
**Triglyceride, mg/dL**	188.8 ± 184.6	121.1 ± 103.9	194.2 ± 188.6	0.106
**HDL cholesterol, mg/dL**	47.4 ± 14.4	53.2 ± 16.0	46.9 ± 14.2	0.075
**LDL cholesterol, mg/dL**	92.0 ± 44.3	116.1 ± 38.2	90.1 ± 44.3	0.016
**eGFR, mL/min/1.73m^2**	103.6 ± 38.5	128.8 ± 60.8	101.6 ± 35.8	0.069
**uACR, mg/g**	122.6 ± 349.7	232.1 ± 631.3	113.7 ± 317.1	0.429
**Insulin, n (%)**	204 (80.6)	18 (94.7)	186 (79.5)	0.106
**ACEi / ARB, n (%)**	108 (42.7)	5 (26.3)	103 (44.0)	0.134
**SGLT2i, n (%)**	63 (24.9)	2 (10.5)	61 (26.1)	0.132
**GLP-1RA, n (%)**	10 (4.0)	0 (0)	10 (4.3)	0.358
**Statin, n (%)**	179 (70.8)	9 (47.4)	170 (72.6)	0.02

Supplementary Table 2. Baseline characteristics according to baseline eGFR

	Total	eGFR ≥60 ml/min per 1.73 m^2	eGFR <60 ml/min per 1.73 m^2	*p*-value
**n (%)**	254	222 (87.4)	32(12.6)	
**Age (years)**	56.7 ± 15.2	55.3 ± 15.1	66.2 ± 11.8	<0.001
**Sex, n(%)**				
**Men**	137 (53.9)	120 (54.1)	17 (53.1)	0.921
**Women**	117 (46.1)	102 (45.9)	15 (46.9)	
**Type of Diabetes, n(%)**				
**Type 1 diabetes**	19 (7.5)	16 (7.2)	3 (9.4)	0.717
**Type 2 diabetes**	235 (92.5)	206 (92.8)	29 (90.6)	
**Diabetes duration (years)**	7.3 ± 8.3	6.3 ± 8.0	14.0 ± 7.1	<0.001
**Systolic BP, mmHg**	128 ± 19.7	127.2 ± 19.4	134.0 ± 20.8	0.089
**Diastolic BP, mmHg**	78.9 ± 12.1	79.3 ± 12.3	76.3 ± 10.7	0.157
**BMI, kg/m^2**	24.7 ± 4.6	24.6 ± 4.6	25.6 ± 3.9	0.223
**Smoking, n (%)**	101 (41.1)	93 (43.3)	8 (25.8)	0.11
**Alcohol, n (%)**	104 (42.3)	96 (44.7)	8 (25.8)	0.136
**Hypertension, n (%)**	139 (54.7)	114 (51.4)	25 (78.1)	0.004
**Cardiovascular disease, n (%)**	48 (19.0)	39 (17.6)	9 (28.1)	0.158
**HbA1c, %**	10.2 ± 2.7	10.1 ±2.7	10.8 ± 2.7	0.162
**Fasting glucose, mg/dL**	187.2 ± 71.3	185.5 ± 68.2	200.6 ± 92.7	0.33
**Fasting Insulin, uIU/mL**	11.4 ± 10.5	11.7 ± 10.8	9.3 ± 6.6	0.288
**HOMA IR**	7.2 ± 29.3	7.5 ± 31.1	4.9 ± 4.9	0.685
**HOMA B**	58.9 ± 173.2	62.3 ± 183.3	32.6 ± 34.2	0.431
**AST, IU/L**	27.8 ± 20.0	28.2 ± 20.2	25.0 ± 18.9	0.394
**ALT, IU/L**	31.7 ± 22.0	32.3 ± 22.0	27.8 ± 22.3	0.281
**Total cholesterol, mg/dL**	179.8 ± 84.5	183.3 ± 88.8	155.3 ± 37.0	0.084

**Triglyceride, mg/dL**	188.8 ± 184.6	191.0 ± 193.7	173.3 ± 103.6	0.619
**HDL cholesterol, mg/dL**	47.4 ± 14.4	48.1 ± 14.7	42.4 ± 10.5	0.01
**LDL cholesterol, mg/dL**	92.0 ± 44.3	94.0 ± 45.1	78.4 ± 35.7	0.068
**eGFR, mL/min/1.73m^2**	103.6 ± 38.5	112.2 ± 32.9	43.7 ± 13.3	<0.001
**uACR, mg/g**	122.6 ± 349.7	78.9 ± 195.2	425.5 ± 786.3	0.019
**Insulin, n (%)**	204 (80.6)	176 (79.6)	28 (87.5)	0.293
**ACEi /ARBs, n (%)**	108 (42.7)	83 (37.6)	25 (78.1)	<0.001
**SGLT2i, n (%)**	63 (24.9)	55 (24.9)	8 (25.0)	0.989
**GLP-1RA, n (%)**	10 (4.0)	9 (4.1)	1 (3.1)	0.797
**Statin, n (%)**	179 (70.8)	150 (67.9)	29 (90.6)	0.008
**CAN, n(%)**				
**Normal**	54 (21.3)	52 (23.4)	2 (6.3)	0.012
**Early**	91 (35.8)	82 (36.9)	9 (28.1)	
**Definite**	109 (42.9)	88 (39.6)	21 (65.6)	

Supplementary Table 3. Baseline characteristics according to baseline uACR

	Total	uACR <30 mg/g	uACR ≥30 mg/g	*p*-value
**n (%)**	254	147 (57.9)	107 (42.1)	
**Age (years)**	56.7 ± 15.2	56.0 ± 15.4	57.7 ± 14.9	0.363
**Sex, n(%)**				
**Men**	137 (53.9)	73 (49.7)	64 (59.8)	0.109
**Women**	117 (46.1)	74 (50.3)	43 (40.2)	
**Type of Diabetes, n(%)**				
**Type 1 diabetes**	19 (7.5)	11 (7.5)	8 (7.5)	1
**Type 2 diabetes**	235 (92.5)	136 (92.5)	99 (92.5)	
**Diabetes duration (years)**	7.29 ± 8.3	6.8 ± 8.6	7.9 ± 7.9	0.285
**Systolic BP, mmHg**	128 ± 19.7	123.4 ± 17.9	134.4 ± 20.3	<0.001
**Diastolic BP, mmHg**	78.9 ± 12.1	77.0 ± 11.3	81.5 ± 12.7	0.004
**BMI, kg/m^2**	24.7 ± 4.6	24.5 ± 4.8	24.9 ± 4.3	0.483
**Smoking, n (%)**	101 (41.1)	55 (39.0)	46 (43.8)	0.697
**Alcohol, n (%)**	104 (42.3)	55 (39.0)	49 (46.7)	0.484
**Hypertension, n (%)**	139 (54.7)	72 (49.0)	67 (62.6)	0.031
**Cardiovascular disease, n (%)**	48 (19.0)	27 (18.5)	21 (19.6)	0.82
**Baseline HbA1c (%)**	10.2 ± 2.7	10.1 ± 2.9	10.3 ± 2.4	0.708
**Fasting glucose, mg/dL**	187.2 ± 71.3	184.3 ± 65.9	191.3 ± 78.5	0.483
**Fasting Insulin, uIU/mL**	11.4 ± 10.5	12.4 ± 12.3	10.0 ± 6.9	0.104
**HOMA_IR**	7.2 ± 29.3	8.7 ± 38.0	4.9 ± 4.0	0.354
**HOMA_B**	58.9 ± 173.2	70.9 ± 222.1	41.7 ± 44.3	0.229
**AST, IU/L**	27.8 ± 20.0	27.1 ± 17.8	28.8 ± 22.8	0.507
**ALT, IU/L**	31.7 ± 22.0	31.1 ± 30.9	32.6 ± 23.6	0.589
**Total cholesterol, mg/dL**	179.8 ± 84.5	181.1 ± 100.1	178.0 ± 57.8	0.783
**Triglyceride, mg/dL**	188.8 ± 184.6	165.5 ± 149.2	219.9 ± 220.2	0.023

**HDL cholesterol, mg/dL**	47.4 ± 14.4	49.5 ± 15.4	44.6 ± 12.4	0.009
**LDL cholesterol, mg/dL**	92.0 ± 44.3	93.0 ± 39.8	90.6 ± 49.8	0.675
**eGFR, mL/min/1.73m^2**	103.6 ± 38.5	106.8 ± 31.5	99.2 ± 46.3	0.122
**uACR, mg/g**	122.6 ± 349.7	9.7 ± 6.2	277.7 ± 499.9	<0.001
**Insulin, n (%)**	204 (80.6)	113 (77.4)	91 (85.0)	0.128
**ACEi / ARBs, n (%)**	108 (42.7)	60 (41.1)	48 (44.9)	0.55
**SGLT2i, n (%)**	63 (24.9)	35 (24.0)	28 (26.2)	0.69
**GLP-1RA, n (%)**	10 (4.0)	6 (4.1)	4 (3.7)	0.881
**Statin, n (%)**	179 (70.8)	101 (69.2)	78 (72.9)	0.521
**CAN, n(%)**				
**Normal**	54 (21.3)	36 (24.5)	18 (16.8)	0.097
**Early**	91 (35.8)	56 (38.1)	35 (32.7)	
**Definite**	109 (42.9)	55 (37.4)	54 (50.5)	

Supplementary Table 4. Correlation test of independent variables to dependent variables

	**eGFR (mL/min/1.73 m2)**		**uACR (mg/g)**	
	Correlation	*p*-value	Correlation	*p*-value
**Age**	-0.560	<0.001	0.018	0.773
**Diabetes Duration**	-0.382	<0.001	0.120	0.057
**Systolic BP**	-0.096	0.128	0.241	<0.001
**Diastolic BP**	0.106	0.092	0.187	0.003
**BMI**	-0.059	0.347	0.021	0.739
**Baseline HbA1c**	0.079	0.212	0.089	0.158
**Fasting glucose**	0.078	0.259	0.020	0.774
**Fasting insulin**	-0.086	0.213	-0.029	0.677
**HOMA_IR**	-0.009	0.900	-0.022	0.754
**HOMA_B**	-0.066	0.336	-0.021	0.765
**AST**	0.017	0.784	-0.076	0.231
**ALT**	0.012	0.844	-0.099	0.118
**Total cholesterol**	0.124	0.052	0.015	0.812
**Triglyceride**	-0.030	0.637	0.137	0.032
**HDL cholesterol**	0.073	0.255	-0.122	0.058
**LDL cholesterol**	0.242	<0.001	-0.023	0.721
**BUN**	-0.680	<0.001	-.232	<0.001
**Cre**	-0.750	<0.001	0.454	<0.001
**eGFR**	1		-0.198	0.002
**uACR**	-0.198	0.002	1	

Correlation between variables is presented as Pearson correlation r.

Supplementary Table 5. Risk factors for reduced eGFR and elevated uACR

		eGFR <60 mL/min/1.73 m^2		uACR ≥ 30mg/g	
		OR (95% CI)	*p-value*	OR (95% CI)	*p-value*
**Age**		1.06 (1.03-1.10)	<0.001	1.01 (0.99-1.03)	0.362
**Men vs. Women**		0.96 (0.46-2.03)	0.921	1.51 (0.91-2.50)	0.11
**Duration of DM**		1.1 (1.06-1.15)	0.001	1.02 (0.99-1.05)	0.284
**BMI**		1.05 (0.97-1.13)	0.223	1.02 (0.97-1.08)	0.481
**Hypertension**		3.38 (1.41-8.15)	0.007	1.74 (1.05-2.90)	0.032
**HbA1c**		1.1 (0.96-1.25)	0.163	1.02 (0.93-1.11)	0.707
**Triglyceride**		1 (1.00-1.00)	0.619	1 (1.00-1.00)	0.034
**HDL**		0.97 (0.94-1.00)	0.041	0.98 (0.96-0.99)	0.01
**ACEi /ARB**		5.94 (2.46-14.33)	<0.001	1.17 (0.71-1.93)	0.55
**Statin**		4.58 (1.35-15.53)	0.015	1.2 (0.69-2.08)	0.521
**CAN**	normal	1(ref)		1(ref)	
	early	2.85 (0.59-13.72)	0.191	1.25 (0.62-2.53)	0.536
	definite	6.2 (1.4-27.53)	0.016	1.96 (1-3.87)	0.051
